# The 14-3-3σ/GSK3β/β-catenin/ZEB1 regulatory loop modulates chemo-sensitivity in human tongue cancer

**DOI:** 10.18632/oncotarget.3896

**Published:** 2015-05-08

**Authors:** Cong Peng, Xiaoting Jia, Yan Xiong, Jiang Yin, Nan Li, Yingen Deng, Kai Luo, Qiong Zhang, Chengkun Wang, Zhijie Zhang, Guopei Zheng, Zhimin He

**Affiliations:** ^1^ Cancer Hospital and Cancer Research Institute of Guangzhou Medical University, Guangzhou 510095, Guangdong, China; ^2^ Department of Pharmacology, Guangzhou Institute of Snake Venom Research, School of Pharmaceutical Sciences, Guangzhou Medical University, Guangzhou 511436, Guangdong, China

**Keywords:** tongue cancer, 14-3-3σ, GSK3β, β-catenin, chemosensitivity

## Abstract

Here we demonstrated that chemotherapy induced 14-3-3σ expression in tongue cancer (TC) cells and overexpressed 14-3-3σ sensitized TC cells to chemotherapy especially in multidrug resistant TC (MDR-TC) cells. In agreement, 14-3-3σ knockdown enhanced resistance of TC cells to chemotherapy. Mechanically, we found 14-3-3σ physically bound to GSK3β in protein level and the binding inhibited β-catenin signaling. Coincidentally, chemotherapy as well as 14-3-3σ overexpression led to increase of GSK3β protein level. Increased GSK3β protein sensitized TC cells to chemotherapy. Moreover, deregulation of 14-3-3σ/GSK3β/β-catenin axis led to overexpressed ZEB1 in TC cells, especially in MDR-TC cells. As a negative feedback loop, ZEB1 bond to *14-3-3σ* promoter to enhance promoter hypermethylation in TC cells. Promoter hypermethylation resulted into the decrease of 14-3-3σ expression. Importantly, a positive correlation was observed between 14-3-3σ and GSK3β protein expression in TC tissues from patients receiving chemotherapy. High levels of 14-3-3σ and GSK3β were associated with better prognosis in TC patients.

## INTRODUCTION

Squamous cell carcinoma (SCC) of the oral cavity represents the tenth most frequent solid cancer worldwide and tongue cancer (TC) is the most common type of oral cancer. In United States alone, it has been estimated 12,060 new cases and 2030 deaths from TC in 2011 [1]. The current treatment options for TC include surgery, radiotherapy and chemotherapy. Chemotherapy mostly based on pingyangmycin (PYM) and/or cisplatin (cDDP), plays an important role in TC treatment and brings many benefits including reducing tumor size, inhibiting distant metastasis and prolonging patient survival [2]. However, in the clinic, many TCs are insensitive to chemotherapy because of the intrinsic and/or acquired drug resistance. Chemo-insensitivity of TCs even correlates with more aggressive cancer behavior and an even worse clinical outcome [3]. In addition, the dose-limiting toxicity of the chemotherapeutic agents prevents their widespread clinical use [4, 5]. Thus, there is an urgent need to fully understand the biological and molecular actions in the response of TC cells to chemotherapy, and to identify new therapeutic targets to improve the efficacy of chemotherapy.

We previously characterized the protein 14-3-3σ in breast cancer cells [6]. 14-3-3σ expression was down-regulated in multidrug resistant breast cancer cells. Restored 14-3-3σ expression sensitized breast cancer cells to chemotherapy via inhibiting Akt activity [7]. 14-3-3σ is a member of 14-3-3 family proteins which is highly conserved over a wide range of mammalian species. 14-3-3σ originally characterized as human mammary epithelium-specific marker 1, is primarily expressed in epithelial cell, and gradually up-regulated during epithelial cell differentiation and senescence [8]. 14-3-3σ has critical roles in signaling transduction and cell cycle regulation, and serves as a target of p53 [9] and BRCA1 [10]. 14-3-3σ can also stabilize p53 protein by blocking MDM2-mediated p53 ubiquitination [11]. Because of its function in cell cycle regulation and p53 protein stability, it is conceivable that 14-3-3σ is a potential tumor suppressor. 14-3-3σ down-regulation has been reported in various types of cancers, including breast [12], gastric [13], lung [14], ovarian [15] and oral cancer [16]. Additionally, overexpressed 14-3-3σ suppresses the anchorage-independent growth of several breast cancer cell lines [17] and inhibits Akt-activated tumorigenicty [18]. Overexpressed 14-3-3σ was also found to inhibit nasopharyngeal carcinoma growth *in vitro* and *in vivo*, and to sensitize cells to chemotherapy induced apoptosis [19]. These investigations strongly suggest that the cancer suppressor function of 14-3-3σ is compromised during tumorigenesis, whereas, the roles and mechanisms of 14-3-3σ in response to chemotherapy have not been fully elucidated. Here, we found PYM or cDDP-based chemotherapy induced 14-3-3σ expression in TC cells, and overexpressed 14-3-3σ sensitized TC cells to chemotherapy via increasing GSK3β protein level and then inactivating β-catenin signaling. Moreover, β-catenin signaling inhibited 14-3-3σ expression by induction of ZEB1 expression.

## RESULTS

### Chemotherapy induces 14-3-3σ expression in tongue cancer cells

**Figure 1:**
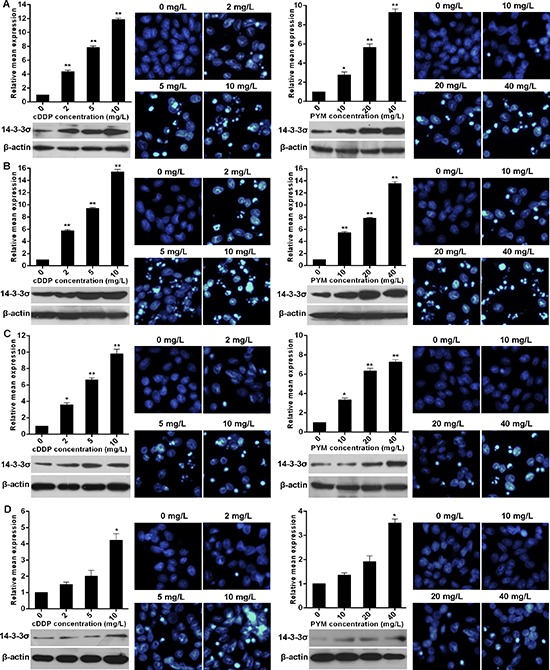
Chemotherapy induces 14-3-3σ expression in tongue cancer cells **A–D.** 14-3-3σ mRNA levels represented as fold change were detected with real-time RT-PCR by normalizing to GAPDH as endogenous control and the expression level from 0 mg/L treatment was set as 1. 14-3-3σ protein levels were detected by western blotting. (A-D) Apoptosis in response to PYM or cDDP treatment for each given cell line was determined by Hoechst staining evaluated by fluorescence microscopy. Cells were collected at 24 h after every treatment for real-time RT-PCR and at 48 h for Hoechst staining. *vs* 0 mg/L treatment, *p < 0.05, **p < 0.01.

To investigate whether 14-3-3σ is involved in chemotherapy response, we firstly detected the expression change of 14-3-3σ upon chemotherapy. We chose two mostly used chemotherapy agents in TC here and found that PYM or cDDP treatment significantly induced 14-3-3σ expression. As shown, both PYM and cDDP dose-dependently induced 14-3-3σ expression in mRNA and protein levels in Tca8113 (Figure 1A), SCC-25 (Figure 1B) and CAL-27 (Figure 1C) TC cell lines respectively. Importantly, the 14-3-3σ induction by chemotherapy was accompanied with apoptosis increase. However, the induction of 14-3-3σ expression in the multidrug resistant TC (MDR-TC) cells Tca8113/PYM established in our previous studies was not so remarkable, accompanied with apoptosis resistance (Figure 1D). Moreover, Tca8113/PYM cell line possesses relatively lower 14-3-3σ expression compared to relatively chemo-sensitive Tca8113, SCC-25 and CAL-27 TC cell lines (Figure 5A). These results strongly implied that 14-3-3σ is potentially involved in chemotherapy response in TC cells.

### 14-3-3σ sensitizes tongue cancer cells to chemotherapy

**Figure 2:**
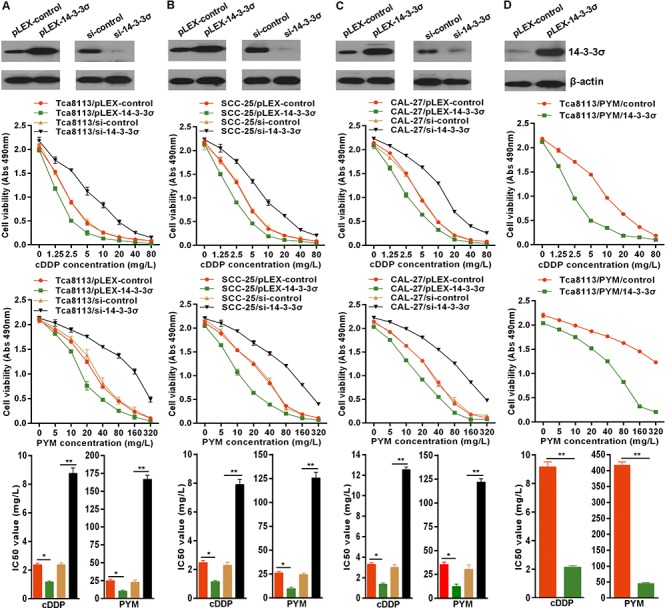
14-3-3σ enhances chemo-sensitivity in tongue cancer cells **A–D.** 14-3-3σ protein levels were detected by western blotting in cells transfected with 14-3-3σ expressing plasmid or siRNA. 14-3-3σ overexpression enhanced the sensitivity of TC cells to PYM and cDDP with reduction of IC50 values, but 14-3-3σ knockdown attenuated the chemo-sensitivity of TC cells with increase of IC50 values detected by MTS proliferation assays. *p < 0.05, **p < 0.01.

14-3-3σ could be induced by chemotherapy, so the role of 14-3-3σ in chemotherapy should be elucidated. Here, we indicated that overexpressed 14-3-3σ via transfection of 14-3-3σ expressing plasmid pLEX-14-3-3σ significantly enhanced the sensitivity of Tca8113 (Figure 2A), SCC-25 (Figure 2B) and CAL-27 (Figure 2C) TC cells to PYM-and cDDP-induced growth inhibition with the marked decrease of IC50 values. Importantly, overexpressed 14-3-3σ also markedly enhanced the sensitivity of MDR-TC cells Tca8113/PYM to chemotherapy with obvious reduction of PYM and cDDP IC50 values (Figure 2D). Inversely, knockdown of 14-3-3σ expression with specific siRNA decrease the sensitivity of Tca8113, SCC-25 and CAL-27 cells to PYM or cDDP, accompanied with significant increase of PYM and cDDP IC50 values (Figure 2A–2C). Results here indicated that the induction of 14-3-3σ expression in chemotherapy is involved in mediating the anti-cancer effects of chemotherapy.

### 14-3-3σ binds to GSK3β protein to inhibit β-catenin activation

**Figure 3:**
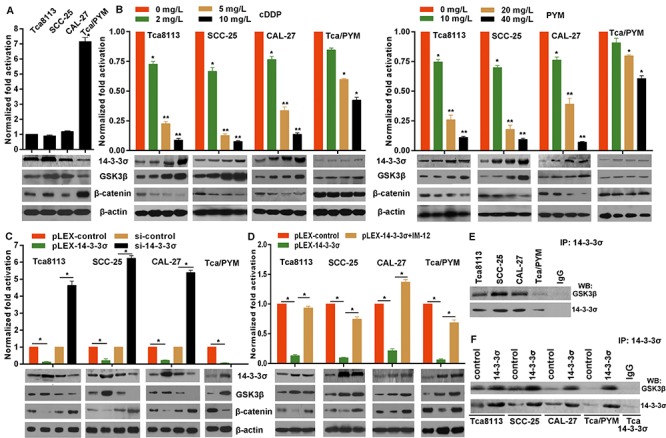
14-3-3σ interacts with GSK3β to inhibit β-catenin signaling activation **A.** The β-catenin signaling activation was determined by TCF/LEF Reporter Assay 48 hours after transfection of related plasmids and the protein levels were detected by western blotting. *vs* other cell lines, *p < 0.01. **B.** Protein levels were detected by western blotting from cells collected 24 h after treatment. 24 h after transfected with related plasmids of TCF/LEF Reporter Assay, cells were treated with PYM or cDDP for 24 h, and then the β-catenin activation was determined. **C.** Protein levels were detected by western blotting from cells collected 48 h after related transfection. The TCF/LEF Reporter Assays were performed in cells at 24 h after transfection with pLEX-14-3-3σ or siRNA, *p < 0.01. **D.** 24 h after cells transfected with pLEX-14-3-3σ or control, the TCF/LEF Reporter Assays were performed. After transfection, all culture media was replaced with fresh media containing 50 nM IM-12. *p < 0.01. **E and F.** Coimmunoprecipitation results demonstrated that 14-3-3σ physically binds to GSK3β and the binding increased with overexpressed 14-3-3σ in TC cells. Equal amounts of cell lysates were immunoprecipitated (IP) with anti-14-3-3σ antibody and then immunoblotted with anti-GSK3β or anti-14-3-3σ bodies, IP with IgG was set as control.

Our previous observations implied the involvement of wnt/β-catenin signaling in chemo-resistance, because the natural wnt/β-catenin antagonist DKK1was down-regulated in MDR-TC Tca8113/PYM cells [20]. Here, the basic β-catenin transactivation was evaluated by TCL/LEF-1 transcriptional activity measured by the TCF/LEF Reporter Assay (luc) Cignal Lenti Reporter Assays (SA Biosciences, Frederick, MD, USA). The results indicated MDR-TC cells Tca8113/PYM possessed higher TCF/LEF-1 transcriptional activity than that in Tca8113, SCC-25 and CAL-27 cell lines (Figure 3A). As a negative regulator of β-catenin, GSK3β protein levels were relatively higher in Tca8113, SCC-25 and CAL-27 cell lines than that in Tca8113/PYM cells. GSK3β protein was inversely correlated with β-catenin protein levels (Figure 3A). To investigate whether 14-3-3σ expression associated with GSK3β and β-catenin protein levels in chemotherapy, we found PYM or cDDP induced marked increase of GSK3β protein levels in Tca8113, SCC-25 and CAL-27 cell lines (Figure 3B) but not in MDR-TC Tca8113/PYM cells. Whereas, chemotherapy induced decrease β-catenin protein levels (Figure 3B) which was inverse with the change of 14-3-3σ and GSK3β protein levels. Directly, we found overexpressed 14-3-3σ attenuated the TCF/LEF-1 transcriptional activity in all selected cell lines (Figure 3C), which was consistent with the change induced by chemotherapy (Figure 3B). However, 14-3-3σ knockdown significantly enhanced the TCF/LEF-1 transcriptional activity (Figure 3C). Because of the important role of GSK3β in β-catenin signaling, we used GSK3β inhibitor IM-12 here and found that IM-12 treatment impaired the effect of overexpressed 14-3-3σ (Figure 3D). 14-3-3σ usually exerts its role via physically interacts with target proteins. To test whether 14-3-3σ could directly bind to GSK3β protein in TC cells, coimmunoprecipitation experiments were performed. We found GSK3β was detected in the anti-14-3-3σ immunoprecipitation complex, and importantly, the overexpressed 14-3-3σ enhanced the binding of 14-3-3σ to GSK3β in selected cell lines (Figure 3E). These findings suggest 14-3-3σ could inhibit β-catenin activation by physically binding to and stabilizing GSK3β protein.

### GSK3β enhances the chemo-sensitivity of tongue cancer cells

**Figure 4:**
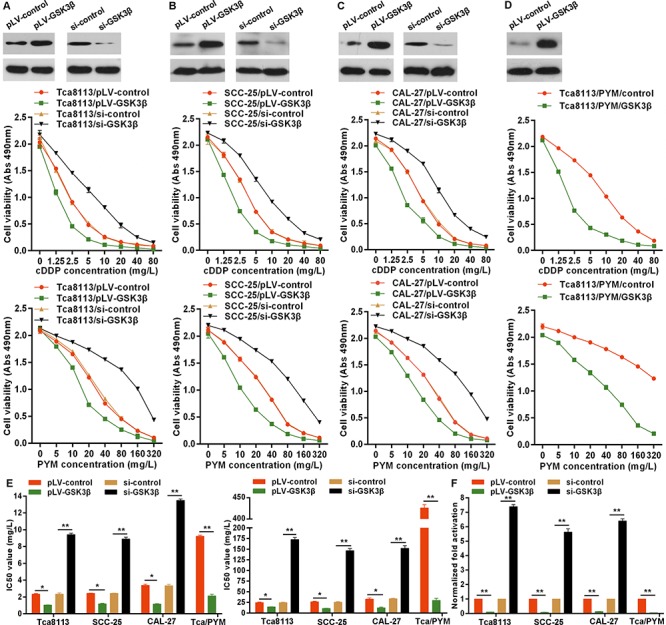
GSK3β enhances chemo-sensitivity in tongue cancer cells **A–E.** GSK3β protein levels were detected by western blotting in cells transfected with GSK3β expressing plasmid or siRNA. GSK3β overexpression enhanced the sensitivity of TC cells to PYM and cDDP with reduction of IC50 values, but GSK3β knockdown attenuated the chemo-sensitivity of TC cells with increase of IC50 values detected by MTS proliferation assays. **F.** 24 h after cells transfected with pLV-GSK3β or siRNA, the TCF/LEF Reporter Assays were performed to determine the effects of GSK3β on β-catenin activation. *p < 0.05, **p < 0.01.

As above observations that GSK3β was involved in the role of 14-3-3σ, we then investigated whether GSK3β was associated with chemo-sensitivity of TC cells. We found overexpressed GSK3β via transfection of GSK3β expressing plasmid pLV-GSK3β enhanced the sensitivity of Tca8113 (Figure 4A), SCC-25 (Figure 4B) and CAL-27 (Figure 4C) cells to PYM-or cDDP-induced growth inhibition with marked decrease of IC50 values (Figure 4E). Especially, overexpressed GSK3β significantly reversed the chemo-resistance of MDR-TC Tca8113/PYM cells to PYM and cDDP (Figure 4D) with remarkable decrease of IC50 values (Figure 4E). However, knockdown of GSK3β with specific siRNA enhanced the resistance of Tca8113 (Figure 4A), SCC-25 (Figure 4B) and CAL-27 (Figure 4C) cell lines to PYM or cDDP with obvious increase of IC50 values (Figure 4E). Mechanically, overexpressed GSK3β attenuated the TCF/LEF-1 transcriptional activity in TC cell lines especially in MDR-TC Tca8113/PYM cells (Figure 4F), whereas, GSK3β knockdown significantly enhanced the TCF/LEF-1 transcriptional activity (Figure 4F). The effect of GSK3β on β-catenin activation was consistent with that of 14-3-3σ.

### ZEB1 enhanced promoter hypermethylation confers to down-regulation of 14-3-3σ in tongue cancer cells

**Figure 5:**
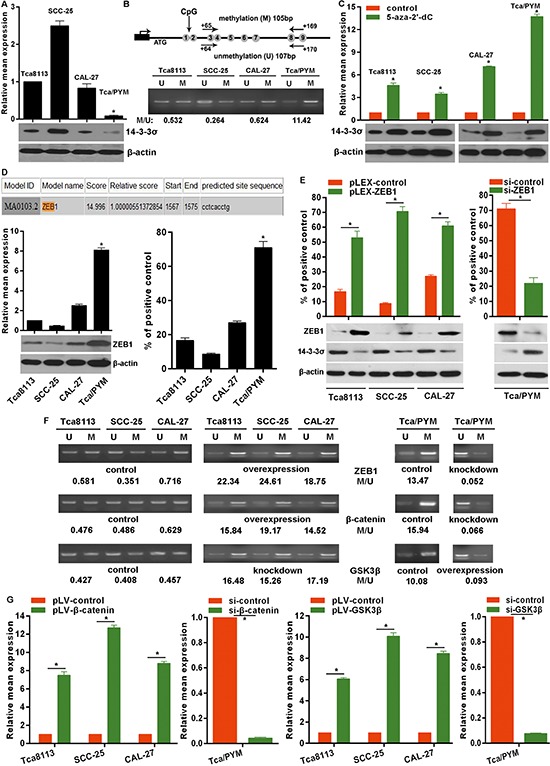
ZEB1 elevates the methylation level of 14-3-3σ promoter **A.** 14-3-3σ mRNA levels represented as fold change were detected with real-time RT-PCR by normalizing to GAPDH as endogenous control and the expression level from Tca8113 was set as 1. 14-3-3σ protein levels were detected by western blotting. **B.** The location of the primers for MSP analysis of *14-3-3σ* gene. MSP analysis determined the *14-3-3σ* promoter methylation levels in TC cell lines. U: unmethylated primers; M: methylated primers. **C.** 14-3-3σ mRNA levels were detected by real-time RT-PCR in TC cells treated with 5 μM 5-aza-2′-dC and the expression level from control-treated cells was set as 1. 14-3-3σ protein levels from related treated cells were detected by western blotting. *vs* control, *p < 0.01. **D.** A schematic representation of ZEB1 binding site in the 2kb putative *14-3-3σ* promoter and the first base of the 2kb strand is defined as ‘1’. ZEB1 mRNA and protein levels were detected by real-time RT-PCR and western blotting respectively. ChIP-qPCR results for the ZEB1 binding to the *14-3-3σ* promoter in TC cell lines. *vs* the other cell lines, *p < 0.01. **E.** ChIP-qPCR results for the ZEB1 binding to the *14-3-3σ* promoter in ZEB1 expression modulated cell lines by transfection of plasmids or siRNA. ZEB1 and 14-3-3σ protein levels were examined by western blotting. **F.** MSP results showed the change of methylation level in the *14-3-3σ* promoter in ZEB1, β-catenin and GSK3β expression modulated cells with transfection of plasmids or siRNAs. **G.** ZEB1 mRNA levels were determined by real-time RT-PCR in β-catenin and GSK3β expression modulated cells by transfection of plasmids or siRNAs. *p < 0.01.

Studies have demonstrated that down-regulation of 14-3-3σ expression is mostly due to DNA hypermethylation rather than gene deletion or mutation [12, 15]. In order to explore whether promoter methylation contributed to the deregulation of 14-3-3σ in TC cells, the methylation status of *14-3-3σ* promoter was examined using MSP (methylation specific PCR). Partial methylation was detected in all selected cell lines, but the methylation level in MDR-TC Tca8113/PYM cells was much higher than that in the other cell lines (Figure 5B), and the methylation level was inversely correlated with 14-3-3σ mRNA and protein levels (Figure 5A). To further demonstrate that promoter hypermethylation is directly responsible for the down-regulation of 14-3-3σ expression, the selected cell lines were subjected to the treatment with 5 μM methyltransferase inhibitor 5-aza-2′-dC for 3 days and then examined for the mRNA and protein expression. After the treatment of 5 μM 5-aza-2′-dC, 14-3-3σ mRNA and protein expression obviously increased, especially in MDR-TC Tca8113/PYM cells (Figure 5C). To determine whether some transcriptional factors regulate the functional state of *14-3-3σ* promoter, we analyzed the response elements of a cohort of transcriptional factors located within a two kilobase region upstream of the first exon of the *14-3-3σ* gene. Using the JASPAR database (http://jaspar.binf.ku.dk) we identified a putative ZEB1 binding sites within this region, conforming to the optimal recognition sequence of ZEB1 (*CACCTG*) (Figure 5D). To confirm the direct association of ZEB1 with the *14-3-3σ* promoter, we performed ChIP-qPCR assays in TC cells for the putative ZEB1 binding site within the two kilobase region. ChIP-qPCR results revealed that ZEB1 could bind to the *14-3-3σ* promoter and the binding level in MDR-TC Tca8113/PYM cells was much higher than that in other TC cell lines (Figure 5D). Expectedly, ZEB1mRNA and protein levels were relatively higher in Tca8113/PYM cells than that in other TC cell lines (Figure 5D). As expected, ectopic expression of ZEB1 using the ZEB1 expressing plasmid pLEX-ZEB1 down-regulated 14-3-3σ mRNA and protein levels (Figure 5E) but enhanced the methylation level of *14-3-3σ* promoter (Figure 5F) in TC cells. Conversely, knockdown of ZEB1 up-regulated 14-3-3σ mRNA and protein levels (Figure 5E) but attenuated the methylation level of *14-3-3σ* promoter in Tca8113/PYM cells (Figure 5F). Moreover, we found that β-catenin exerted the same molecular effects on *14-3-3σ* promoter as ZEB1, but GSK3β exerted the opposite effects (Figure 5F). Furthermore, we demonstrated that ZEB1 expression increased following overexpression of β-catenin in TC cells, but ZEB1expression decreased following β-catenin knockdown in Tca8113/PYM cells using β-catenin-specific siRNA, which was opposite from the results of interference of GSK3β expression (Figure 5G). These results indicated that β-catenin and GSK3β regulated 14-3-3σ expression via ZEB1 and that ZEB1 binding enhances the *14-3-3σ* promoter hypermethylation to suppress 14-3-3σ expression in TC cells.

### 14-3-3σ and GSK3β are positive prognostic markers in tongue cancer

**Figure 6:**
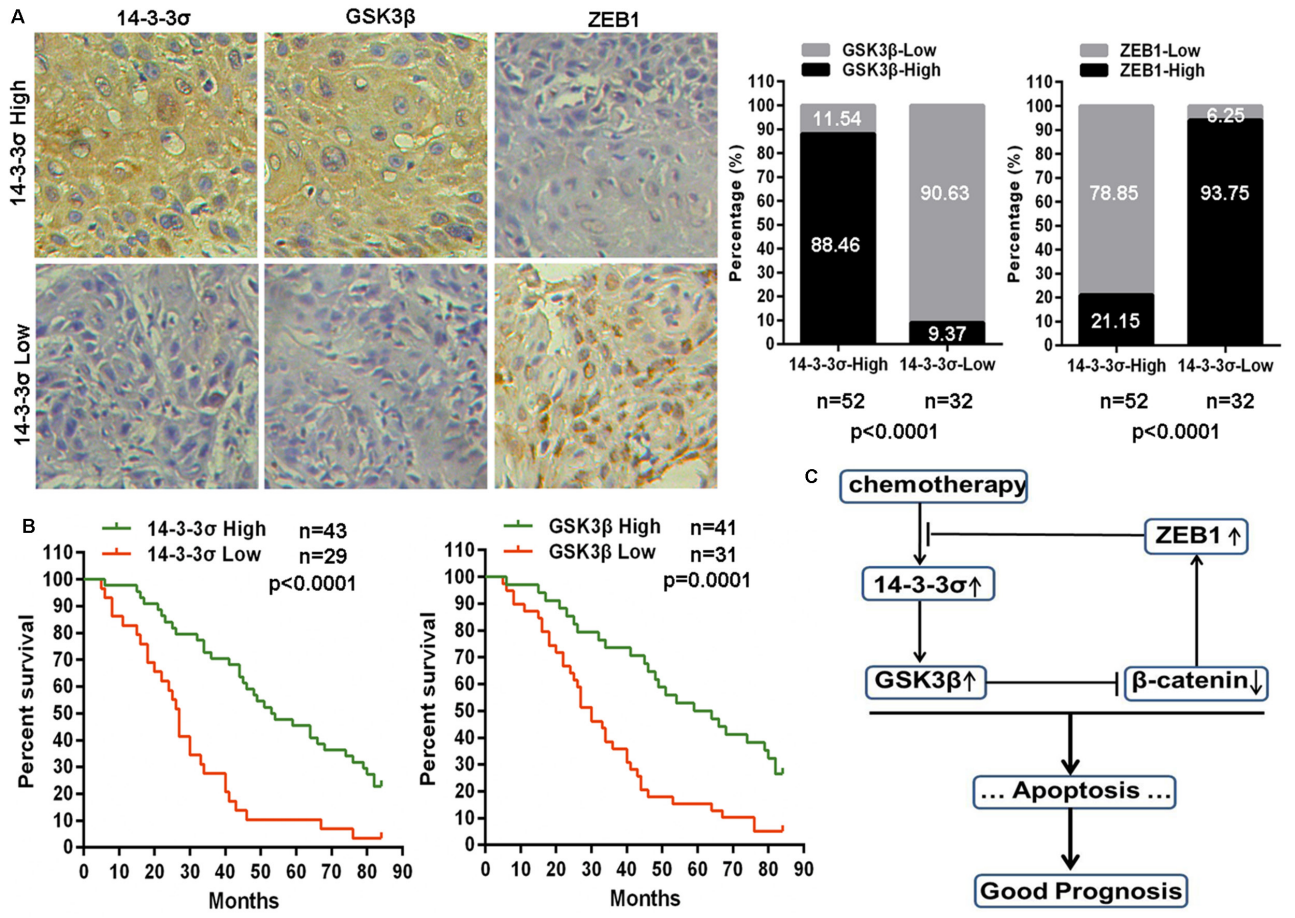
14-3-3σ correlates with GSK3β and ZEB1 in tongue cancer and is associated with good clinical prognosis **A.** Representative images of 14-3-3σ, GSK3β and ZEB1 protein levels detected by immunohistochemical staining in tongue cancer tissues (20×). 14-3-3σ protein level was positively correlated with GSK3β protein level, but was negatively correlated with ZEB1 protein level in tongue cancer tissues. **B.** Kaplan-Meier analysis estimated overall survival according to the 14-3-3σ protein level and GSK3β protein level in tongue cancer patients. **C.** Schematic model depicting the 14-3-3σ/GSK3β/β-catenin/ZEB1 feedback regulatory loop in chemotherapy response of tongue cancer.

Given that chemotherapy induces 14-3-3σ expression to stabilize GSK3β protein in TC cells, and that 14-3-3σ and GSK3β sensitizes TC cells to chemotherapy, we next wished to assess whether there is a correlation between 14-3-3σ and GSK3β in TC tissues, and whether they are valuable prognostic markers for TC patients. We therefore performed immunohistochemical staining on TC tissues from 84 patients receiving chemotherapy based on PYM and/or cDDP. High 14-3-3σ protein levels were found in 52 tissues, of which 46 tissues were stained with high GSK3β protein levels (Figure 6A). In contrast, GSK3β protein was highly expressed in only 3 of 32 TC tissues that exhibited low 14-3-3σ protein level upon chemotherapy (Figure 6A). Additionally, of the 52 tissues with high 14-3-3σ protein levels, 41 tissues possessed low ZEB1 protein levels (Figure 6A). But of the 32 tissues with low 14-3-3σ protein levels, 30 tissues expressed high ZEB1 protein levels (Figure 6A). Importantly, patients with highly expressed 14-3-3σ protein upon chemotherapy had significantly longer survival times (Figure 6B). GSK3β also is a positive prognostic marker for tongue cancer patients (Figure 6B). All the results denote a potentially positive correlation between 14-3-3σ and GSK3β protein levels but a potentially negative correlation between 14-3-3σ and ZEB1 protein levels, and that elevated 14-3-3σ and GSK3β levels upon chemotherapy predict better treatment outcomes in people with tongue cancer.

## DISCUSSION

Although significant advances have been achieved in the treatment of various types of cancer including tongue cancer, the clinical outcome still remains unsatisfactory. This underlines the critical importance of elucidating the precise molecular events involved in chemotherapy response and identifying new effective targets to improve current treatments in the clinic. In the present study, we found that chemotherapy markedly induced 14-3-3σ expression in mRNA and protein levels dose-dependently, accompanied with apoptosis increase in TC cell lines. However, chemotherapy failed to induce 14-3-3σ expression in the multidrug-resistant TC cells. Actually, 14-3-3σ mRNA and protein levels were much lower in drug-resistant TC cells than that in relative drug-sensitive TC cell lines. Ectopic overexpression of 14-3-3σ sensitized TC cells to chemotherapy, especially in the multidrug-resistant cells, whereas, knockdown of 14-3-3σ expression led to the decrease of chemo-sensitivity. The role of 14-3-3σ as a tumor suppressor has been reported because of its down-regulation and critical roles in signal transduction in various types of cancers [9–19].

In order to explore the mechanisms responsible for 14-3-3σ sensitizing TC cells to chemotherapy, we focused on GSK3β/β-catenin signaling, because our previous data suggested activation of GSK3β/β-catenin signal in multidrug resistant tongue cancer (MDR-TC) cells Tca8113/PYM [20]. Expectedly, 14-3-3σ expression was inversely correlated with the activation of β-catenin in TC cells, represented as GSK3β protein decrease and β-catenin protein increase in MDR-TC cells. Chemotherapy significantly induced GSK3β protein increase, but β-catenin protein decrease dose-dependently in TC cells, but not in MDR-TC cells. Overexpressed 14-3-3σ inhibited β-catenin activation, but 14-3-3σ knockdown remarkably enhanced β-catenin activation in TC cells. Interestingly, GSK3β inhibitor impaired the effect of 14-3-3σ on β-catenin inactivation, suggesting GSK3β mediated the role of 14-3-3σ. Actually, GSK3β sensitized TC cells to chemotherapy. Mechanically, our data strongly suggested 14-3-3σ physically interacted with GSK3β to stabilize GSK3β protein. GSK3β plays a key role within the APC complex in initiating proteasomal degradation of β-catenin. When GSK3β function is inhibited, protein stabilization of β-catenin allowing it to translocate to the nucleus to bind with TCF/LEF transcription factors and then to drive the transcriptional activation of genes involved in cell growth, survival, invasion, metastasis and the stem characteristics [21]. Deregulation of GSK3β protein usually led to β-catenin signaling activation, such as h-prune interact with GSK3β and promotes sequestration of GSK3β inside multivesicular bodies to induce activation of β-catenin signaling [22]. β-catenin is a well-known oncogene present in many cancers, which is involved in promoting carcinogenesis [23], supporting cancer cells survival [24], modulating metastasis [25] and inducing drug resistance and cancer stem cell phenotype [26].

Given that chemotherapy obviously induced 14-3-3σ expression in transcriptional levels in relative drug-sensitive tongue cancer cells but not in MDR-TC cells, and that as a potential tumor suppressor 14-3-3σ is reported to be frequently down-regulated in various types of cancer due to promoter hypermethylation, we detected the promoter methylation status of *14-3-3σ* in selected tongue cancer cell lines. Methylated *14-3-3σ* promoter could be detected in all the four cell lines especially in MDR-TC cells. Treatment with 5 μM 5-aza-2′-dC greatly up-regulated 14-3-3σ mRNA and protein levels especially in MDR-TC cells, implying aberrant methylation is directly responsible for the aberrant expression of 14-3-3σ in tongue cancer cells. To explore which molecules maintain the methylated status of *14-3-3σ* promoter, we identified ZEB1 physically binds to the *14-3-3σ* promoter and enhanced the DNA hypermethylation. ZEB1 protein level positively correlated with the methylation level in TC cells. Overexpressed ZEB1 enhanced the promoter methylation level and 14-3-3σ down-regulation, but ZEB1 knockdown attenuated the promoter methylation level and promote the expression of 14-3-3σ in TC cells. ZEB1 has been reported to be highly expressed in epithelial cancers and its expression correlates with poor prognosis [27]. ZEB1 is a known driver of epithelial-to-mesenchymal transition (EMT), a phenotype associated with cancer cells that are typically prone to metastasis, drug resistance and poor clinical outcome. ZEB1 has been reported tightly associated with cancer initiation, invasion, chemo-resistance and radio-resistance in various types of cancers [28, 29]. A few reports also have indicated the association between ZEB1 and DNA methylation, such as defective binding of ZEB1 results into hypomethylation which contributes to increased constitutive levels of p73 [30]. Additionally, ZEB1 can down-regulate E-cadherin expression via recruiting histone deacetylases HDAC1 and HDAC2 in pancreatic cancer [31]. But whether and how ZEB1 recruits DNA methyltransferases to enhance DNA methylation levels to modulate genes expression remains to be explored. The effect of ZEB1 mediated methylation change was accordant with β-catenin done, but was opposite from the effect of GSK3β. In addition, we found β-catenin positively regulated ZEB1 expression, but GSK3β showed a converse effect on ZEB1 expression in TC cells. Consistently, β-catenin has been reported to transactivate ZEB1 expression to promote cancer invasion and metastasis [32, 33], of which the detailed mechanisms will be elucidated in our further studies.

Taken together, as shown in Figure 6C, we demonstrated an important role of GSK3β/β-catenin signaling in modulating the effects of 14-3-3σ on chemo-sensitivity in TC cells. As a negative feedback mechanism, β-catenin/ZEB1 enhances the methylation level of *14-3-3σ* promoter to down-regulate 14-3-3σ expression in TC cells. Our findings strongly suggest that 14-3-3σ can be a prognostic biomarker for tongue cancer patients and new strategies with 14-3-3σ administration may significantly enhance the efficacy of chemotherapy against human tongue cancer.

## MATERIALS AND METHODS

### Cell culture and tissue specimens

The moderately differentiated human tongue squamous cell carcinoma derived cell line Tca8113 was obtained from the China Center for Type Culture Collection (Wuhan, China) and the stable multidrug-resistant cell line Tca8113/PYM was previously established by induction with PYM in our lab. The squamous cell carcinoma cell lines SCC-25 and CAL-27 were from American Type Culture Collection. Above cell lines were cultured in RPMI-1640 (Gibco, Carlsbad, CA, USA) containing 10% fetal bovine serum (Gibco) at 37°C in a humidified atmosphere containing 5% CO_2_. To maintain the resistance phenotype, 0.5 mg/L PYM was added to the culture media of Tca8113/PYM cells. PYM was from PYM Harbin Bolai Pharmaceutical (Harbin, China). cDDP and 5-aza-2′-dC were from Sigma-Aldrich (Steinheim, Germany). MI-12 was from Selleck Chemicals (Houston, Texas, USA). Eighty-four tongue cancer tissue specimens were obtained from patients at the Affiliated Cancer Hospital of Guangzhou Medical University between March 2000–December 2006. Overall survival was computed from the day of surgery to the day of death or of last follow-up. The study was approved by the ethics committee of the Affiliated Tumor Hospital of Guangzhou Medical University.

### Real-time PCR for mature miRNAs and mRNAs

The total RNA was extracted according to the Trizol protocol, and cDNAs from the mRNAs were synthesized with the first-strand synthesis system (Thermo Scientific, Glen Brunie, MA, USA). Real-time PCR was carried out according to standard protocols using an ABI 7500 with SYBR Green detection (Applied Biosystems, Foster City, CA, USA). GAPDH was used as an internal control and the qRT-PCR was repeated three times. The primers for GAPDH were: forward primer 5′-ATTCCATGGCACCGTCAAGGCTGA-3′, reverse primer 5′-TTCTCCATGGTGGTGAAGACGCCA-3′; primers for 14-3-3σ were: forward primer 5′-ACTTTTCCGTCTTCCACTACGA-3′, reverse primer 5′-ACAGTGTCAGGTTGTCTCGC-3′; primers for ZEB1 were: forward primer 5′-GATGATGAATGCGAGTCAGATGC-3′, reverse primer 5′-ACAGCAGTGTCTTGTTGTTGT-3′.

### Hoechst staining

Cells were seeded in fresh medium in 24-well plates. After a 24 h incubation, cells were treated with or without cDDP or PYM at different concentrations for an additional 48 h. The cells were then stained with hoechst 33528, and apoptotic cells possessing significantly smaller, condensed and fragmented nuclei, were observed using a fluorescence microscope.

### Cells transfection

Cells were trypsinized, counted and seeded into six-well plates the day before transfection to ensure 70% cell confluency on the day of transfection. The transfection of the pLEX-14-3-3σ, pLV-GSK3β, pLV-β-catenin, pLEX-ZEB1 vectors and related controls was carried out using Lipofectamine 2000 (Invitrogen, Carlsbad, CA, USA) in accordance with the manufacturer's instructions. siRNAs targeting 14-3-3σ, GSK3β, β-catenin or ZEB1 and siRNA controls were purchased from Santa Cruz Biotechnology (Dallas, Texas, USA). Transfection of siRNA (50 nM final concentration) was performed as above. Experiments were performed 48 h post-transfection.

### MTS assay

The CellTiter 96 AQueous One Solution Cell Proliferation Assay kit (Promega, Madison, WI, USA) was used to determine the sensitivity of cells to cDDP or PYM. Briefly, cells were seeded in 96-well plates at a density of 4 × 10^3^ cells/well (0.2 ml/well) for 24 h before use. The culture medium was replaced with fresh medium containing cDDP or PYM at different concentrations and cells were then incubated for a further 72 h. Then, MTS (0.02 ml/well) was added. After a further 2 h incubation, the absorbance at 490 nm was recorded for each well on the BioTek Synergy 2. The absorbance represented the cell number and was used for the plotting of dose-cell number curves and then IC50 values were calculated.

### Coimmunoprecipitation

For the co-immunoprecipitation assay, the cells were lysed with modified TNE buffer (50 mM Tris [pH 8.0], 150 mM NaCl, 1% Nonidet P-40 [NP-40], 10 mM sodium fluoride, 10 mM sodium pyrophosphate, 2 mM EDTA) supplemented with 1 mg/L leupeptin, 1 mg/L aprotinin, and 1 mM sodium orthovanadate (Na_3_VO_4_). The immunoprecipitations were performed overnight at 4°C with antibodies to 14-3-3σ or IgG (as a control). The immunoprecipitates were then incubated for 2 h with protein G-agarose (Amersham Biosciences, Piscataway, NJ, USA). The reaction products were washed with lysis buffer, and the immune complexes were resolved by SDS-PAGE. Subsequently, western blots were performed.

### TCF/LEF reporter assay

Activity of β-catenin signaling was determined as luciferase transcription dependent on T-cell factor/lymphoid enhancer factor (TCF/LEF) with the Cignal Reporter Assay Kit (SA Biosciences, Frederick, MD, USA). Lipofectamine 2000 (Invitrogen, Carlsbad, CA, USA) was used to transfect cells per well in 96-well plates with 100 ng of the TCF/LEF reporter plasmid. Resulting firefly and Renilla luciferase activities were determined with the DualGlo Luciferase Assay System (Promega). All conditions were measured in triplicates and repeated in an independent experiment. Functionality and transfection efficiency were controlled using the negative and positive control plasmid contained within the Cignal Reporter Assay Kit.

### Methylation specific PCR (MSP)

DNA was extracted from cells using a DNA kit (Tiangen, Beijing, China). Bisulfite treatment and PCR amplification were performed as previously described with some modifications [34]. In brief, 2 μg genomic DNA was denatured in sodium hydroxide (NaOH) (0.2 N) for 15 min at 37°C before adding 30 μl hydrochinone (20 μM, Sigma) and 520 μl sodium bisulfite (3 M, pH 5.0) which converts unmethylated but not methylated cytosines to uracil. The samples were mixed and incubated at 50°C for 16 h. Modified DNA was purified with the Wizard DNA purification resin (Promega), treated with 6.3 μl NaOH (5N) at room temperature for 5 min to complete the conversion, precipitated with ethanol and resuspended in water. Methylation-specific PCR was performed with primers specific for either methylated or the modified unmethylated DNA spanning the region between CpG dinucleotides 3 and 9 within the 14-3-3σ gene (Figure 5B). The primers specific for methylated DNA were the following: 5′-TGGTAGTTTTTATGAAAGGCGTC-3′ (sense) and 5′-CCTCTAACCGCCCACCACG-3′ (antisense); the primers specific for unmethylated DNA were the following: 5′-ATGGTAGTTTTTATGAAAGGTGTT-3′ (sense) and 5′-CCCTCTAACCACCCACCACA-3′ (antisense), which yielded 105-bp and 107-bp PCR products, respectively. The PCR conditions were as follows: 1 cycle of 95°C for 5 min; 31 cycles of 95°C for 45 s, 56°C for 30 s and 72°C for 30 s; and 1 cycle of 72°C for 4 min. The PCR samples were resolved by electrophoresis in a 2% agarose gel and stained with ethidium bromide.

### Western blotting

Total protein was extracted from cells using RIPA buffer (Thermo Scientific, Rockford, IL, USA) in the presence of protease inhibitors (Protease Inhibitor Cocktail, Thermo Scientific). The protein concentration of lysates was measured using a BCA Protein Assay Kit (Thermo Scientific). Equivalent amounts of protein were mixed with 5 × Lane Marker Reducing Sample Buffer (Thermo Scientific), and resolved by electrophoresis in a 10% SDS–polyacrylamide gel and then transferred onto Immobilon-P Transfer Membrane (Merck Millipore, Schwalbach, Germany). The membranes were blocked with 5% non-fat milk in Tris-buffered saline and then incubated with primary antibodies followed by secondary antibody. The signal was detected on the Odyssey instrument (LI-COR Bioscience, Lincoln, Nebraska USA). 14-3-3σ and GSK3β antibodies were from Santa Cruz Biotechnology (Dallas, Texas, USA). B-catenin, ZEB1 and β-Actin antibodies were from Cell Signaling Technology (Danvers, MA, USA). The fluorescently labeled secondary antibodies were from LI-COR Bioscience.

### ChIP-qPCR

The ChIP assay was performed using the EZ-CHIP™ chromatin immunoprecipitation kit (Merck Millipore). Briefly: Chromatin proteins were cross-linked to DNA by addition of formaldehyde to the culture medium to a final concentration of 1%. After a 10 min incubation at room temperature, the cells were washed and scraped off in ice-cold phosphate-buffered saline (PBS) containing Protease Inhibitor Cocktail II. Cells were pelleted and then resuspended in lysis buffer containing Protease Inhibitor Cocktail II. The resulting lysate was subjected to sonication to reduce the size of DNA to approximately 200–1000 base pairs in length. The sample was centrifuged to remove cell debris and diluted ten-fold in ChIP dilution buffer containing Protease Inhibitor Cocktail II. Then 5 μg of anti-RNA Polymerase antibody (positive control, included with the kit), or anti-ZEB1 antibody (cell signal technology) were added to the chromatin solution and incubated overnight at 4°C with rotation. After antibody incubation, protein G agarose was added and the sample incubated at 4°C with rotation for an additional 2 h. The protein/DNA complexes were washed with Wash Buffers four times and eluted with ChIP Elution Buffer. Cross-links were then reversed to free DNA by the addition of 5 M NaCl and incubation at 65°C for 4 h. The DNA was purified according to the manufacturer's instructions. 50 μl of DNA was obtained for each treatment. 2 μl of DNA from each group was used as a template for PCR. Primers for the *14-3-3σ* promoter containing putative ZEB1 binding sites were as follows, sense: 5′-GGTGGGGATTAAATTCGATCG-3′, antisense: 5′-GAGCCCATAAAGGTTCAGGAG-3′ (for site A); Primers for the human GAPDH gene: sense, 5′-TACTAGCGGTTTTACGGGCG-3′, antisense, 5′-TCGAACAGGAGGAGCAGAGAGCGA-3′. The PCR conditions were as follows: 1 cycle of 95°C for 5 min; 40 cycles of 95°C for 20 s, 60°C for 30 s, and 72°C 30 s; and 1 cycle of 72°C for 10 min. The results were calculated by normalizing to the positive control, and relative quantization values were calculated using % positive control = 2^(−ΔCt [(Ct [14-3-3σ] − (Ct [positive control]]) method.

### Immunohistochemistry

Human tongue cancer specimens were cut into 4-μm sections. The sections were dried at 62°C for 2 h and then deparaffinized in xylene and rehydrated using a series of graded alcohol washes. The tissue slides were then treated with 3% hydrogen peroxide in methanol for 15 min to quench endogenous peroxidase activity and antigen retrieval then performed by incubation in 0.01 M sodium cirate buffer (pH 6.0) and heating using a microwave oven. After a 1 h preincubation in 10% goat serum, the specimens were incubated with primary antibody overnight at 4°C. The tissue slides were treated with a non-biotin horseradish peroxidase detection system according to the manufacturer's instruction (DAKO, Glostrup, Denmark). Two different pathologists evaluated the immunohistological samples. The intensity of immunostaining was taken into consideration when analyzing the data. The intensity of staining was scored from 0 to 3 and the expression was classified as high if the score was ≥2, and as low if the score was ≤1.

### Statistical analysis

All statistical analyses were performed with SPSS statistical software (version 21.0; IBM). Survival curves were constructed using the Kaplan–Meier method and analyzed by the log-rank test. Significant prognostic factors identified by univariate analysis were entered into multivariate analysis using the Cox proportional hazards model. The Student's *t*-test was used for comparisons and the Pearson correlation test (two-tailed) was used to investigate the correlation between 14-3-3σ and GSK3β or ZEB1 protein levels. Statistical significance was defined as *p* < 0.05.
